# Implementation strategies to improve preconception and antenatal care for tobacco smoking, alcohol consumption and weight management: a systematic review protocol

**DOI:** 10.1186/s13643-019-1193-3

**Published:** 2019-11-23

**Authors:** Emma Doherty, Melanie Kingsland, Luke Wolfenden, John Wiggers, Julia Dray, Jenna Hollis, Elizabeth J. Elliott, Justine B. Daly, Kylie A. Bailey, John Attia, Mandy Hunter, Ian Symonds, Belinda Tully, Danika Tremain, Rebecca K. Hodder

**Affiliations:** 1 Hunter New England Population Health, Hunter New England Local Health District, Locked Bag 10, Wallsend, New South Wales 2287 Australia; 2 School of Medicine and Public Health, The University of Newcastle, Callaghan, New South Wales Australia; 3 Hunter Medical Research Institute, New Lambton Heights, New South Wales Australia; 4 Faculty of Medicine and Health and Discipline of Child and Adolescent Health, University of Sydney, Sydney, New South Wales Australia; 5 Sydney Children’s Hospital Network, Kids’ Research Institute, Westmead, New South Wales Australia; 6 Hunter Primary Care, Newcastle, New South Wales Australia; 7 Maternity and Gynaecology John Hunter Hospital, New Lambton Heights, New South Wales Australia; 8 Adelaide Medical School, The University of Adelaide, Adelaide, South Australia Australia

**Keywords:** Implementation, Antenatal, Preconception, Modifiable risk factors

## Abstract

**Background:**

Despite existing best practice care recommendations for addressing tobacco smoking, alcohol consumption and weight management in preconception and antenatal care, such recommendations are often not implemented into routine practice. Effective strategies that target known barriers to implementation are key to reducing this evidence to practice gap. The aim of this review is to synthesise the evidence on the effectiveness of implementation strategies in improving the provision of preconception and antenatal care for these modifiable risk factors.

**Methods:**

Randomised and non-randomised study designs will be eligible for inclusion if they have a parallel control group. We will include studies that either compare an implementation strategy to usual practice or compare two or more strategies. Participants may include any health service providing preconception or antenatal care to women and/or the health professionals working within such a service. The primary outcome will be any measure of the effectiveness of implementation strategies to improve preconception and/or antenatal care for tobacco smoking, alcohol consumption and/or weight management (including care to improve nutrition and/or physical activity). Secondary outcomes will include the effect of the implementation strategy on women’s modifiable risk factors, estimates of absolute costs or cost-effectiveness and any reported unintentional consequences. Eligible studies will be identified via searching Cochrane Central Register of Controlled Trials, MEDLINE, EMBASE, Maternity and Infant Care, CINAHL, ProQuest Dissertations and Theses and other sources (e.g. contacting experts in the field). Study selection, data extraction and risk of bias will be assessed independently by two review authors and differences resolved by a third reviewer. If data permits, we will conduct fixed-effects or random-effects meta-analysis where appropriate. If studies do not report the same outcome or there is significant heterogeneity, results will be summarised narratively.

**Discussion:**

This review will identify which implementation strategies are effective in improving the routine provision of preconception and antenatal care for tobacco smoking, alcohol consumption and weight management. Such a review will be of interest to service providers, policy makers and implementation researchers seeking to improve women’s modifiable risk factors in preconception and antenatal care settings.

**Systematic review registration:**

PROSPERO CRD42019131691

## Background

The presence of modifiable risk factors prior to conception and during pregnancy can have significant implications for pregnant women and their babies [1]. Three of the most prevalent modifiable risk factors that can adversely impact pregnancy and offspring outcomes are tobacco smoking, alcohol consumption and gestational weight gain outside of recommended ranges (including inadequate nutrition and physical activity) [1]. Internationally, it is estimated that during pregnancy: 10% of women smoke tobacco [2–4], 10% consume alcohol [5] and 68% gain weight outside of recommended ranges [1, 6–8]. However, these rates vary considerably and reported prevalence in some countries and population groups is much higher [1]. Each of these modifiable risk factors is associated with an increased risk of pregnancy complications and poor obstetric outcomes, including spontaneous abortion, small or large for gestational age, preterm birth and need for neonatal intensive care [6, 9–12]. Further negative impacts of these risk factors include poor infant and child outcomes, such as developmental delay and obesity, which can have long term consequences and increase the risk of chronic diseases in adulthood [6, 9, 13–15]. Clustering of these modifiable risk factors prior to and during pregnancy is also well established, which can increase such risks through cumulative effects [16–18].

Timely access to health care prior to pregnancy (preconception care) and during pregnancy (antenatal or prenatal care) contributes to better maternal and child health outcomes and fewer clinical interventions [1, 19]. Clinical guidelines provide best practice care recommendations for health professionals who see women prior to and during pregnancy [1, 20–23]. Such guidelines recommend that as part of routine preconception and antenatal care, all women are universally assessed for tobacco smoking, alcohol consumption and weight; provided advice; and offered targeted support (e.g. counselling, brief intervention or pharmaceutical support) if required [1, 20–23]. As part of weight management care, it is further recommended that women receive advice and appropriate support for nutrition and physical activity [1, 20–23].

Such guidelines are supported by systematic review evidence that indicates interventions are effective in reducing these risk factors prior to and during pregnancy. For example, psychosocial interventions are effective in increasing smoking cessation during pregnancy [24]; psychological, educational and brief interventions are effective in reducing alcohol consumption and increasing alcohol abstinence during pregnancy [25, 26]; and educational and behavioural interventions targeting nutrition and/or physical activity are effective in preventing excessive gestational weight gain [27, 28]. Preconception care may also be effective in improving risk factors prior to pregnancy [8], including lowering rates of risky alcohol consumption [29].

Despite the existence of clinical guideline recommendations and evidence for interventions addressing modifiable risk factors in preconception and antenatal settings, many women do not routinely receive such best practice care [30]. For example, a study of 1173 women in the UK reported low levels of receipt of preconception advice from general practitioners on tobacco smoking (13%), alcohol consumption (13%) and healthy weight (10%) [31]. An Australian study of 223 pregnant women found that the majority of women reported being asked about smoking (97%) and alcohol (92%) during their antenatal care, but less than half (48%) reported having their weight gain assessed [32]. Of those women who reported requiring further support to manage their risks, 62% were offered assistance for smoking, 10% for alcohol consumption and 36% for weight management [32]. With these varying levels of care provision, clinical guideline recommendations designed to improve pregnancy outcomes are unlikely to achieve their intended benefits and, as such, strategies are needed to reduce the current evidence to practice gap in guideline care.

Implementation frameworks recommend that system and individual level barriers to care provision need to be identified so that appropriate behaviour change techniques are applied when selecting strategies to improve practice [33]. Numerous barriers have previously been reported to impede health professional’s provision of care for tobacco smoking, alcohol consumption and weight management in preconception and antenatal care settings, including lack of supporting systems, resources and time within the consult [34–43]; lack of knowledge of the risk factors and care procedures [38, 43]; lack of skills and confidence in delivering care to women and limited training opportunities to address this [35, 36, 39, 41, 43, 44]; and a reluctance to ask women about their health risks due to a perception that it will have a negative effect on the client-clinician relationship [43]. Such barriers present a considerable challenge for health professionals and managers seeking to improve guideline implementation in these settings [33].

A number of systematic reviews have reported on the effectiveness of implementation strategies in improving care when similar barriers are present in health care settings more broadly, including prompts and system reminders [45], educational meetings and materials [46, 47], educational outreach visits [48, 49], local opinion leaders [50–52] and audit and feedback [53]. Specific to the antenatal setting, one previous review has reported on the effectiveness of strategies in increasing smoking cessation care [54] and another has reported on health provider focussed interventions to support obese pregnant women [55], with the latter review identifying no eligible studies. Despite tobacco smoking, alcohol consumption and weight gain outside of recommended ranges often co-occurring in pregnant women, and preconception and antenatal guideline recommendations and reported barriers to care provision being similar across these modifiable risk factors, no reviews to date have synthesised the evidence for the effectiveness of implementation strategies in increasing preconception and antenatal care across these modifiable risk factors.

## Objective

The objective of this review is to determine the effectiveness of implementation strategies in improving the routine provision of preconception and/or antenatal care for tobacco smoking, alcohol consumption and/or weight management (including care to improve nutrition and/or physical activity) to women.

## Methods

The systematic review has been registered with the International Prospective Register of Systematic Reviews (PROSPERO; registration number: CRD42019131691). This review protocol was reported in accordance with the Preferred Reporting Items for Systematic Reviews and Meta-Analyses Protocol (PRISMA-P) recommendations [56] (see Additional file 1).

### Eligibility criteria

#### Types of studies

Non-randomised and randomised study designs with a parallel control group will be eligible for inclusion. Non-randomised study designs will be included due to the challenges of using randomised designs for complex public health and system focussed interventions [57]. Eligible study designs include the following:

- Randomised studies (e.g. randomised controlled studies, randomised cluster studies, randomised staggered enrolment or stepped-wedge studies);
- Non-randomised studies (e.g. non-randomised controlled studies, non-randomised cluster studies, non-randomised staggered enrolment or stepped-wedge studies);
- Controlled before-after studies (CBAs) and cluster CBAs; and
- Interrupted time-series studies that have independent control groups.

We will only include studies that (1) compare an implementation strategy that seeks to improve preconception and/or antenatal care for tobacco smoking, alcohol consumption and/or weight management (including care to improve nutrition and/or physical activity) with no intervention or ‘usual practice’, or (2) compares two or more implementation strategies that seek to improve preconception and/or antenatal care for these risk factors. There will be no restrictions on the length of the study follow up period, country of origin, language of publication or year of study.

#### Participants

Participants will be services and health professionals responsible for delivering preconception or antenatal care in public or privately operated settings such as primary care, hospital maternity care, specialist medical services, midwifery services or family planning services. Health professionals could include but are not limited to general practitioners, family physicians, obstetrician-gynaecologists, fertility specialists, midwives or nurses. Studies in settings that do not usually provide care to women prior to or during pregnancy, such as community education campaigns, will be excluded.

#### Implementation strategies

Studies that specifically aim to improve care for the selected risk factors using one or more implementation strategies will be included. Implementation strategies could include, but are not limited to, those described in the Cochrane Effective Practice and Organisation of Care (EPOC) and Expert Recommendations for Implementing Change (ERIC) taxonomies, including clinical practice guidelines, educational meetings, educational materials, local opinion leaders, record system changes, reminders, audit and feedback and monitoring performance [58, 59]. Interventions may be a single strategy (e.g. point of care reminder in a medical record system) or multi-strategy (e.g. provision of educational materials and local opinion leaders).

#### Outcomes

The primary outcome will be any measure of the effectiveness of implementation strategies to improve preconception and/or antenatal care for tobacco smoking, alcohol consumption and/or weight management (including care to improve nutrition and/or physical activity).

For example, the proportion of health professionals who ask women about their smoking after a system reminder is implemented or the mean number of occasions women report receiving advice on alcohol consumption throughout their pregnancy after health professionals receive educational materials. Data from self-report measures (e.g. by health professionals or women), direct observation by researchers, audits of medical records (e.g. patient pregnancy records) or other methods will be eligible.

Secondary outcomes will include the following:

1. Effect of the implementation strategy on women’s tobacco smoking, alcohol consumption, weight, gestational weight gain, nutrition and/or physical activity prior to or during pregnancy. Data could include self-report or physical measurements (e.g. weight gain).
2. Estimates of absolute costs or the cost-effectiveness of the implementation strategies to improve preconception and antenatal care for tobacco smoking, alcohol consumption and/or weight management.
3. Any reported unintentional consequence of the implementation strategy (e.g. impacts on staff attitudes or changes to women’s antenatal care schedules).

### Search methods

We will perform searches for eligible peer-reviewed and grey literature studies in electronic databases, and a range of other sources.

#### Electronic sources

The following electronic bibliographic databases will be searched:

- Cochrane Central Register of Controlled Trials (CENTRAL, current issue);
- MEDLINE (including MEDLINE in Process, ePub Ahead of Print and other non-indexed citations), Ovid (1946 to present);
- EMBASE, Ovid (1947 to present);
- Maternity and Infant Care, Ovid (1985 year to present);
- CINAHL, EBSCOhost (1980 to present); and
- ProQuest Dissertations and Theses.

#### Other sources

Searches will also be undertaken in the following sources:

- Reference lists of all included studies for citations of other potentially eligible studies;
- Hand searching of all publications for the past three years in the journals *Implementation Science*, *Journal of Translational Behavioural Medicine*, *BMC Pregnancy* and *Childbirth and Midwifery*;
- World Health Organization International Clinical Trials Registry Platform (www.who.int/trialsearch/);
- Google (first 200 results); and
- Experts in the field and key organisations will be contacted and other relevant websites searched to identify any other potentially eligible studies.

#### Search strategy

The strategy will include search terms for participant, implementation strategies, study design and outcomes. Modified search filters published in previous Cochrane systematic reviews for implementation strategies [60, 61] and study design [61, 62] will be utilised. A validated search filter for non-randomised study designs will not be used, which may be a limitation to the strategy. The MEDLINE search strategy is described in Additional file 2. It will be adapted for other databases using appropriate syntax and terminology in consultation with a Research Librarian.

### Data collection and analysis

#### Study selection

Two review authors will independently screen titles and abstracts identified through the search strategy described above. Review authors will not be blind to author or journal information as per the Cochrane Handbook, which acknowledges there is uncertainty that blinding protects against author bias [63]. Studies that do not meet the review eligibility criteria based on the initial title and abstract screen will be excluded. Two review authors will independently review the full text of all remaining studies for eligibility. Study authors will be contacted for clarification for any studies where there is insufficient information to determine eligibility. Where sufficient information is unavailable to determine eligibility, the study will be excluded from the review. The primary reason for exclusion will be recorded for all full-text studies. Abstracts in any language other than English will be translated using Google Translate. Any discrepancies in title and abstract or full-text screening will be resolved by consensus or a third reviewer if required. Study selection will be managed through Covidence.

#### Data extraction

Two review authors will independently extract information from the eligibile studies. Authors extracting data will not be blind to author or journal information as per the Cochrane Handbook, which acknowledges there is uncertainty that blinding protects against author bias [63]. Data will be extracted using a standardised form that will be adapted from previous systematic reviews undertaken by the review team [60, 61]. The form will be piloted prior to use. Study authors will be contacted for additional information about the characteristics of the strategies implemented where limited detail is provided. Any discrepancies regarding data extraction will be resolved by consensus or a third reviewer if required.

The following information will be extracted:

- Study characteristics: authors, date of publication, country of study, aim of study, setting (preconception or antenatal), participant characteristics (service and/or health professional type), study design, number of experimental conditions and information to assess risk of bias.
- Implementation strategy characteristics: strategy type (to allow classification against the EPOC taxonomy [58]), theoretical underpinning of the strategy, duration of the implementation strategy, implementation strategy dose (e.g. number of training sessions), implementation strategy reach (e.g. number of clinicians who received training), implementation strategy fidelity (e.g. extent to which training was delivered to protocol) and external contextual factors that may have impacted on strategy implementation (e.g. change in guidelines or risk factor recommendations).
- Study primary and secondary outcomes: data collection method, name of tool or system, validity of measures used, scale of measure, number of participants per comparison group at each time point, effect size and measures of outcome variability.
- Cost or cost effectiveness of the intervention.
- Any unintentional positive or negative consequences of the implementation strategy (e.g. changes in staff attitudes or changes to antenatal schedules).
- Sources of funding and any potential conflicts of interest.

#### Assessment of risk of bias

Two review authors will independently assess risk of bias in randomised study designs using the Cochrane Risk of Bias Tool [63]. Each of the following domains will be assigned a ‘high’, ‘low’ or ‘unclear’ bias classification: (1) random sequence generation (selection bias), (2) allocation concealment (selection bias), (3) blinding of participants and personnel (performance bias), (4) blinding of outcome assessment (detection bias), (5) incomplete outcome data (attrition bias), (6) selective outcome reporting (reporting bias) and (7) any other potential sources of bias. For cluster randomised study designs, the following additional criteria will be assessed: recruitment to cluster, baseline imbalance, loss of clusters, incorrect analysis and compatibility with individually randomised controlled studies [63]. For non-randomised study designs, two review authors will independently assess the following risk of bias criteria using the Newcastle-Ottawa Scale (NOS): (1) selection, (2) comparability and (3) outcome [64]. Any discrepancies will be resolved by consensus or a third reviewer with expertise in review methodology if required.

#### GRADE

Two review authors will independently assess the overall quality of the evidence for each of the primary outcomes using the GRADE approach [65] with any disagreements to be resolved by consensus or a third reviewer if required. The GRADE quality ratings (from ‘very low’ to ‘high’) will be used to describe the body of evidence with randomised and nonrandomised designs presented separately. Randomised studies will start from a high rating and non-randomised studies will start from a low rating.

#### Measures of treatment effect

It is anticipated that differences in the types of interventions in included studies may preclude the use of summary statistics to describe the treatment effect. This may necessitate findings being presented in narrative form. Nonetheless, outcome data will be synthesised using meta-analyses where possible and appropriate to do so. In such cases, the standard estimation of the risk ratio (RR) and 95% confidence intervals (CI) will be calculated for dichotomous outcomes. For continuous outcomes, mean differences (MDs; where consistent outcome measures are reported) or standardised mean differences (SMDs; where different outcome measures are reported) and 95% CIs will be calculated.

#### Data synthesis

Clinical heterogeneity will first be assessed to determine whether it is appropriate to combine results in a meta-analysis. If it is deemed that studies cannot be combined in a meta-analysis, a narrative synthesis will be presented. If it is deemed that studies can be combined in a meta-analysis, on the basis of Cochrane Handbook guidance [63], a fixed-effects model will be adopted in the first instance if studies are sufficiently homogenous and RR, MD or SMDs will be calculated. However, if there is evidence of high heterogeneity, a random effects model will be utilised instead. Data from randomised and non-randomised study designs will be synthesised separately.

Where studies report outcomes using different data collection methods or scales, the one that is judged by the authors to represent the most valid measure will be used for data synthesis. For studies that include multiple intervention or control arms, only the arms that meet the eligibility criteria will be included. In cases where multiple arms are included, a decision will be made to either (1) collapse all intervention and/or control arms into single pairwise comparisons or (2) conduct bivariate analyses with all eligible arms included and adjust for the repeated inclusion of the same intervention and/or control arm [63]. In studies with multiple follow up points, the data collection point measured furtherest from recruitment will be analysed.

#### Unit of analysis issues

For cluster studies, individual level data that adjusts for clustering will be extracted. In studies where the effects of clustering have not been adjusted for, study authors will be contacted to provide intraclass correlation coefficients (ICCs). Where ICCs are not available, a mean ICC will be estimated from included studies with similar outcomes and used to calculate effective sample sizes.

#### Dealing with missing data

The proportion of participants lost to follow up will be reported and considered in the risk of bias assessment as potential evidence of attrition bias. Any instances whereby sensitivity analyses have been conducted by study authors using different assumptions to deal with missing data will be recorded. Reported data that has adopted the intention to treat (ITT) principle will be extracted in preference to study data that does not. If an included study has no such ITT data, the data that is available will still be extracted.

#### Assessment of heterogeneity

Characteristics of studies will be considered for intervention and methodological heterogeneity. If required, visual inspection of forest plots will be undertaken to inspect statistical heterogeneity. If studies are deemed to be sufficiently homogenous based on these initial inspections, heterogeneity for each outcome will be statistically quantified by calculating the *I*^2^ statistic with a cut-point of > 50% to be indicative of substantial heterogeneity [63]. Decisions to perform meta-analysis will be based on discussions between study authors following consideration of these measures of heterogeneity.

#### Assessment of reporting biases

Published studies will be compared to protocols and registers (where available) to identify instances of potential selective reporting within studies. If meta-analyses are deemed appropriate and there are at least 10 studies included, funnel plots will be generated for each outcome to determine potential publication bias.

#### Sensitivity analysis

If there are sufficient studies, sensitivity analyses for the primary outcome will be conducted by removing studies with an overall high risk of bias to examine their impact on the effect estimate.

## Discussion

This systematic review will synthesise current evidence for the effectiveness of implementation strategies in improving the routine provision of preconception and antenatal care for tobacco smoking, alcohol consumption and/or weight management (including care to improve nutrition and/or physical activity). Such a review will be of benefit to services providing preconception and antenatal care, policy makers and implementation researchers with an interest in reducing the gap between the evidence base and clinical practice for the prevention of adverse outcomes due to maternal tobacco smoking, alcohol consumption and gestational weight gain outside of recommended ranges.

## Supplementary information

**Additional file 1:** PRISMA-P 2015 Checklist (pdf file).

**Additional file 2:** Medline Search Strategy (pdf file).

## Data Availability

Not applicable.

## Abbreviations

CBA: Controlled before and after
CI: Confidence interval
EPOC: Effective Practice and Organisation of Care
ERIC: Expert Recommendations for Implementing Change
ITT: Intention to treat
MDs: Mean differences
NOS: Newcastle-Ottawa scale
RR: Risk ratio
SMDs: Standardised mean differences
