# Supplementary material for: Implementation strategies to improve preconception and antenatal care for tobacco smoking, alcohol consumption and weight management: a systematic review protocol
Source: Syst Rev. 2019 Nov 23;8:285. doi: 10.1186/s13643-019-1193-3 (PMC6874816; doi:10.1186/s13643-019-1193-3)
Supplement: Supplementary file 2 — Additional file 2: Medline Search Strategy (pdf file). [file 13643_2019_1193_MOESM2_ESM.docx]

Implementation strategies to improve preconception and antenatal care for tobacco smoking, alcohol consumption and weight management

MEDLINE Search Term Strategy

1. Pregnancy/ or Pregnan*.tw.

2. Matern*.tw.

3. Gestation*.tw.

4. Preconcept*.tw.

5. Trying to conceive.tw.

6. 1 or 2 or 3 or 4 or 5

7. Midwifery/ or Midwi*.tw. or Nurse Midwives/

8. Obstetric*.tw. or Obstetrics/

9. Physicians, Family/ or Family Practice/ or General Practic*.tw. or General Practice/ or General Practitioners/

10. clinician*.tw.

11. ((health or healthcare) adj2 (profession* or work*)).mp.

12. Prenatal Care/ or (antenatal or prenatal).tw.

13. Preconception Care/

14. Perinatal Care/ or perinatal.tw.

15. Maternal Health Services/ or Maternity.tw. or Family Planning Services/

16. (family planning or fertility specialist*).tw.

17. 7 or 8 or 9 or 10 or 11 or 12 or 13 or 14 or 15 or 16

18. implement*.mp.

19. dissemin*.mp.

20. adopt*.mp.

21. practice*.mp.

22. organi?ational change*.mp.

23. diffus*.mp.

24. (system* adj2 change*).mp.

25. quality improvement*.mp.

26. transform*.mp.

27. translat*.mp.

28. transfer*.mp.

29. uptake*.mp.

30. sustainab*.mp.

31. institutionali*.mp.

32. routin*.mp.

33. maintenance.mp.

34. capacity.mp.

35. incorporat*.mp.

36. adher*.mp.

37. integrat*.mp.

38. scal*.mp.

39. ((polic* or guideline or practice* or program* or innovate*) adj5 (performance or feedback or audit* or monitor* or academic detailing or prompt* or reminder* or medical record* or record system* or incentive* or penalt* or mandat* or communicat* or social market* or professional development or network* or leadership* or opinion leader* or champion* or consensus* or change manage* or train* or educat* or resource* or material* or equipment or guideline)).mp.

40. 18 or 19 or 20 or 21 or 22 or 23 or 24 or 25 or 26 or 27 or 28 or 29 or 30 or 31 or 32 or 33 or 34 or 35 or 36 or 37 or 38 or 39

41. Fetal Alcohol Spectrum Disorders/

42. Alcohol Abstinence/ or Alcohol*.tw.

43. Drinking behaviour/ or Alcohol Drinking/ or Drinking/ or drink*.tw. or Binge Drinking/

44. Ethanol.tw. or Ethanol/

45. (Smok* adj2 (prevent* or reduc* or cessation or cease* or cigarette or tobacco)).mp.

46. Tobacco/ or Tobacco Smoking/ or Tobacco.tw.

47. Cigarette*.tw. or Tobacco Products/

48. Smoking Cessation/ or Nicotine Replacement.tw. or NRT.tw. or Smoking/

49. Weight gain/ or Weight gain.tw.

50. Nutri*.tw.

51. Diet.tw. or Diet/ or Healthy Diet/

52. (Food.tw. or Food/) and Nutrition/

53. Eat*.tw. or Eating/

54. Energy Intake.tw. or Energy Intake/

55. Physical Activit*.tw.

56. Exercise/ or Exercise.tw.

57. Sedentary Lifestyle/ or Physical Inactivit*.tw.

58. (Sedentary adj2 (Behavio* or Lifestyle)).mp.

59. Fitness.tw. or Physical Fitness/

60. 41 or 42 or 43 or 44 or 45 or 46 or 47 or 48 or 49 or 50 or 51 or 52 or 53 or 54 or 55 or 56 or 57 or 58 or 59

61. Randomized Controlled Trial/

62. Controlled Clinical Trial/

63. Clinical Trials as Topic/

64. Random Allocation/

65. Evaluation Studies/

66. Comparative Study/

67. random*.tw.

68. trial.tw.

69. groups.tw.

70. placebo.tw.

71. experiment*.tw.

72. (time adj series).tw.

73. (pretest or pre test or posttest or post test).tw.

74. impact.tw.

75. change*.tw.

76. evaluat*.tw.

77. effect*.tw.

78. 'before and after'.tw.

79. intervention*.tw.

80. program*.tw.

81. compare*.tw.

82. (control or controls* or controla* or controle* or controli or controll*).tw.

83. (Stepped wedge or staggered enrol*).tw.

84. 61 or 62 or 63 or 64 or 65 or 66 or 67 or 68 or 69 or 70 or 71 or 72 or 73 or 74 or 75 or 76 or 77 or 78 or 79 or 80 or 81 or 82 or 83

85. Ask*.tw.

86. Screen*.tw. or Mass Screening/

87. Assess*.tw. or Risk Assessment/

88. Advi?e.tw. or Health Education/

89. Assist*.tw.

90. Arrang*.tw.

91. (Refer*.tw. or Referral.mp.)

92. brief intervention.tw. or Motivational Interviewing/ or Psychotherapy, Brief/

93. 5A*.tw.

94. SBIRT.tw.

95. ((Care or practi?e*) adj (best or evidence* or recomm*)).tw.

96. 85 or 86 or 87 or 88 or 89 or 90 or 91 or 92 or 93 or 94 or 95

97. 6 and 17 and 40 and 60 and 84 and 96
